# Is Current Social Distancing Enough?

**DOI:** 10.1007/s10439-021-02741-y

**Published:** 2021-02-11

**Authors:** Sasa Kenjeres, Frank S. Henry, Akira Tsuda

**Affiliations:** 1 Department of Chemical Engineering, Faculty of Applied Sciences, Delft University of Technology, 2629 HZ Delft, The Netherlands; 2 Department of Mechanical Engineering, Manhattan College, Riverdale, NY 10471 USA; 3 Tsuda Lung Research, Shrewsbury, MA 01545 USA

To the editor,

The separation distance recommended^2^ to lower the risk of being infected by SARS COV-2 virions via airborne transmission^3^ appears to be largely based on data from large-droplet experiments in quiescent environments.^6^ Here, we investigate how far exhaled airflow during normal speech can transport smaller particles^5^ into quiescent air and into an environment with ambient air motion.

We modeled expiratory flow during normal conversational speech as a jet of constant velocity of 1 m/s and 10 s in duration^4^ through an elliptical orifice. We seeded the expiratory airflow with SARS COV-2 droplet nuclei (≈ 4 *μ*m in diameter^5^) and considered the jet’s development in quiescent air (Fig. 1a) and in an environment with a low-speed tailwind 1 (Fig. 1b). In both cases, the simulation showed that while the jet was smooth, axisymmetric, and fully laminar at the mouth, it eventually became unstable and turbulent; however, significant differences in the details of the flows are evident. In the quiescent case, transition occurred a few orifice diameters from the mouth, and once turbulent, further forward motion of the jet was impeded (Fig. 1a). In the tailwind case, the laminar region persisted much longer, and the turbulent cloud was transported farther by the ambient air motion (Fig. 1b). (also, please see accompanied animations.)

**Figure 1:**
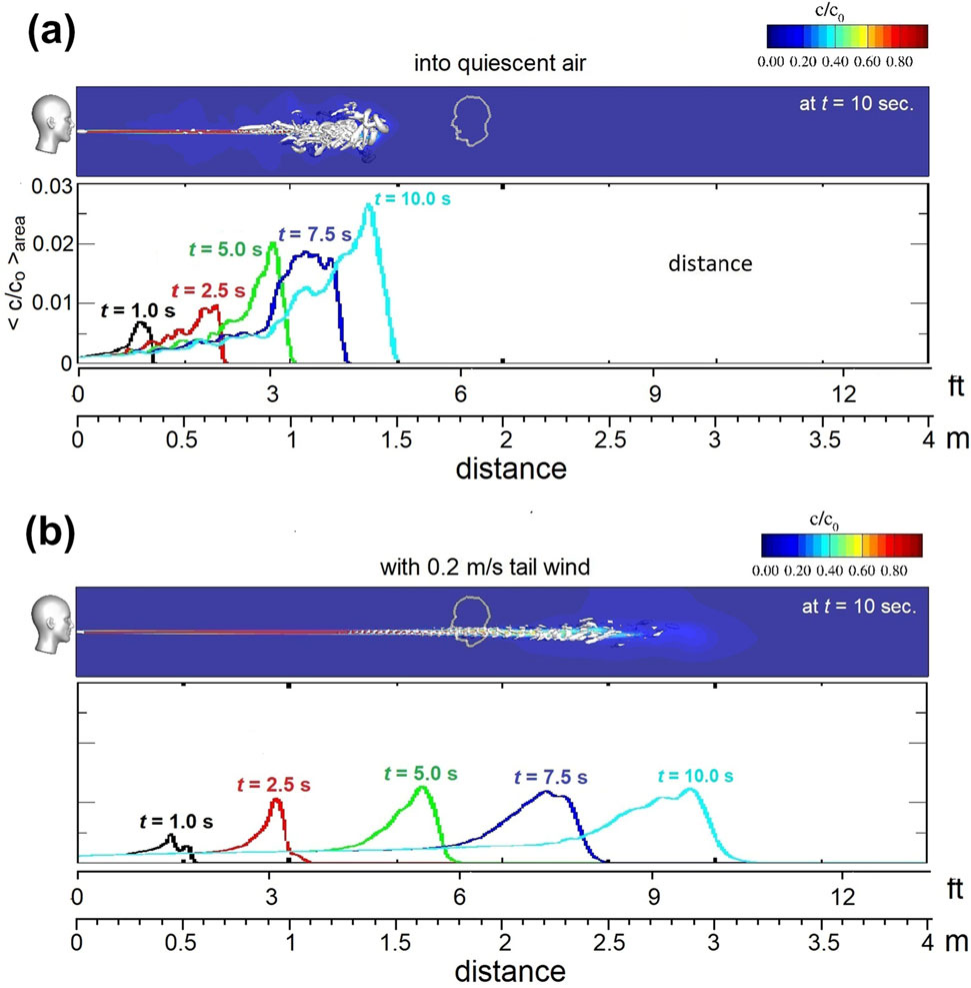
The trajectory of virus-laden^3^ exhaled air while speaking for 10 s in two different environments (quiescent air vs. a low-speed tailwind). The grayscale, cloud-like, structures shown in the upper panels denote eddy structures/local vortical flow structures, and the color-maps shown in the lower panels give the local cross-sectional average concentration (*c*/*c*_o_; where *c*_o_ is the concentration at the mouth) over time in the central vertical plane. Respiration physiology during speaking is different from that of tidal breathing. Inspiration vs. expiration ratio is about 1:9, instead of approximately 1:1 during normal breathing. After a rapid inhalation of air, the lung volume (usually ~ 35% of vital capacity) decreases nearly linearly during speech. A linear decrease of lung volume implies a constant expiratory flow. A mouth opening was modeled as an orifice of elliptic shape, whose area is ~ 1.8 cm^2^ (an average value for an adult) with an aspect ratio of 0.6. This results an orifice flow rate of 1 m/s. We simulated the flow and local concentration of droplets using Large Eddy Simulation (LES), which is based on solving discretized forms of conservation of mass, momentum and concentration of species (Eulerian approach, with 2nd order accurate finite-volume based TU Delft in-house computer code). The entire simulation domain is represented by approximately 4 million (Nx:Ny:Nz = 402:102:102) non-uniformly distributed hexagonal control volumes covering a total simulation domain of 4.0 × 0.42 × 0.42 m^3^. The simulation time-step was 0.01 s.

While the quiescent case (Fig. 1a) supports the social distancing recommendation of six feet^2^ (≈ 1.8 m), much caution should be applied. In practice, the air is unlikely to be still, and our investigation reveals that the reach of the virus-laden^3^ exhaled air is strongly influenced by the ambient airflow. Also, we modeled normal speech (with ~1 m/s expiratory flow rate) but the further people are from each other, the louder they tend to speak. Speaking more loudly or choiring results in an increase in both exhaled and inhaled air volume, and both could promote an increase in virus transmission.^3^

It is also important to differentiate between outside and indoor gathering. Although it is likely that any virus-laden^3^ exhaled air is quickly diluted by the ambient air in the case of outside gatherings, indoor gatherings could be fertile grounds for virus transmission. We conclude that the distance virus-laden^3^ exhaled air travels during speech depends strongly on the motion of the ambient air. While the ambient air motion considered here would be barely perceptible,^1^ we have shown that even this small air current can double the reach of the virus-laden^3^ air.

## Supplementary Information

Below is the link to the electronic supplementary material.

Supplementary material 1 (GIF 2722 kb)

Supplementary material 2 (GIF 2497 kb)
