# Supplementary figures and images for: Is Current Social Distancing Enough?
Source: Ann Biomed Eng. 2021 Feb 11;49(9):1973–4. doi: 10.1007/s10439-021-02741-y (PMC7878170; doi:10.1007/s10439-021-02741-y)

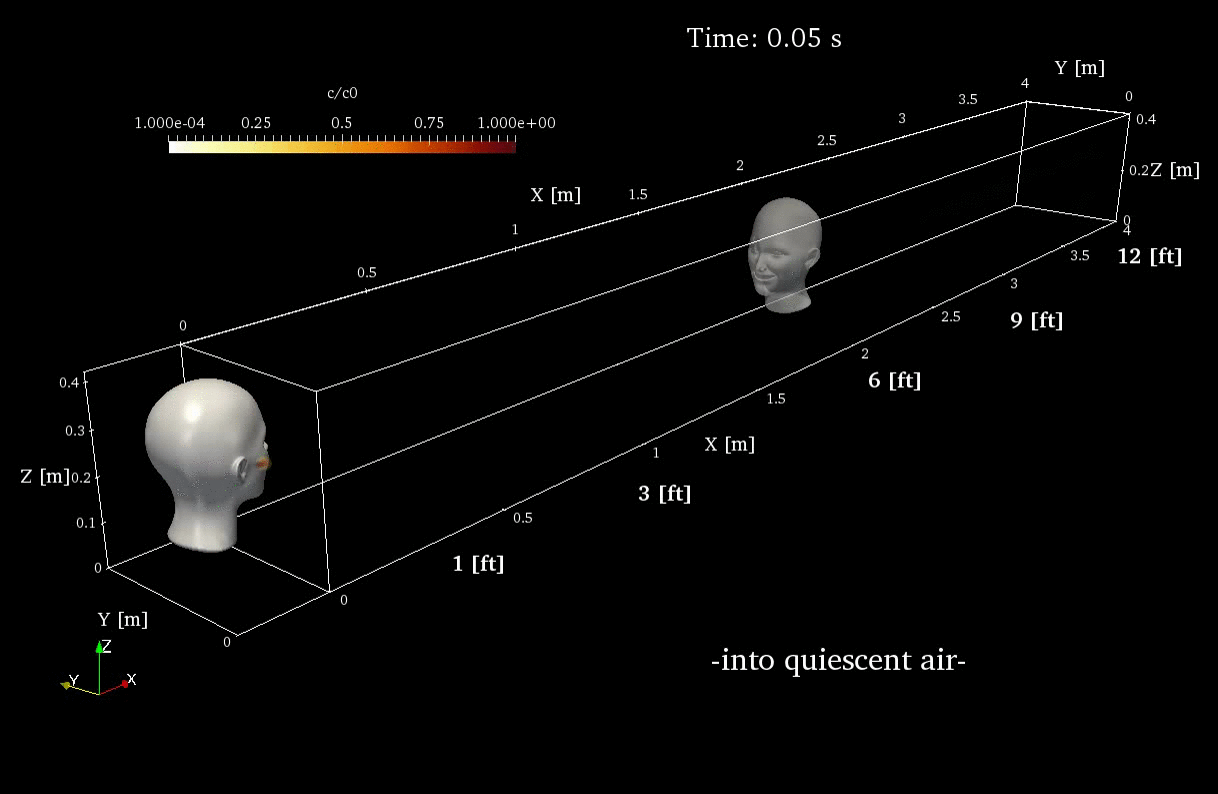

Supplement: Supplementary file 1 — Supplementary material 1 (GIF 2722 kb) [file 10439_2021_2741_MOESM1_ESM.gif]

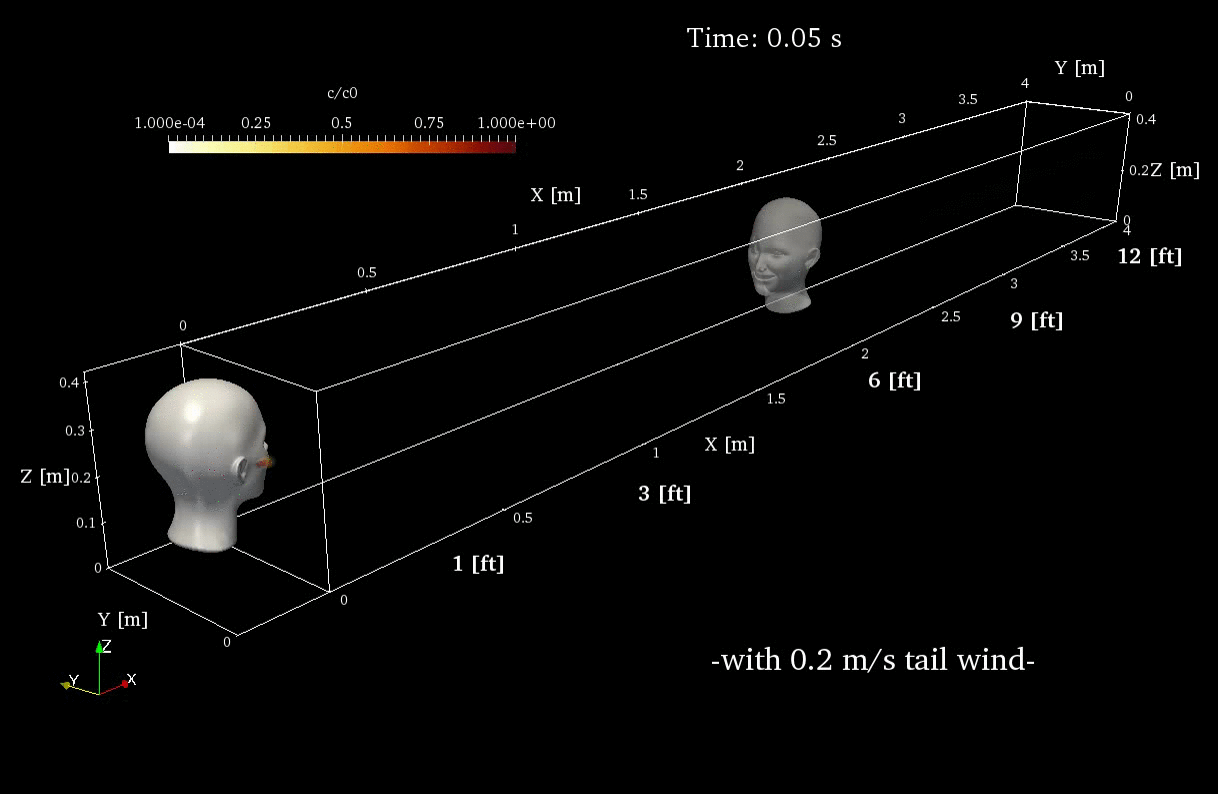

Supplement: Supplementary file 2 — Supplementary material 2 (GIF 2497 kb) [file 10439_2021_2741_MOESM2_ESM.gif]
